# Diversity of Extended *HLA-DRB1* Haplotypes in the Finnish Population

**DOI:** 10.1371/journal.pone.0079690

**Published:** 2013-11-21

**Authors:** Annika Wennerström, Efthymia Vlachopoulou, L. Elisa Lahtela, Riitta Paakkanen, Katja T. Eronen, Mikko Seppänen, Marja-Liisa Lokki

**Affiliations:** 1 Transplantation Laboratory, Haartman Institute, Helsinki, Finland; 2 Division of Infectious Diseases, Department of Medicine, Helsinki University Central Hospital, Helsinki, Finland; Centro Cardiologico Monzino IRCCS, Italy

## Abstract

The Major Histocompatibility Complex (MHC, 6p21) codes for traditional HLA and other host response related genes. The polymorphic *HLA-DRB1* gene in MHC Class II has been associated with several complex diseases. In this study we focus on MHC haplotype structures in the Finnish population. We explore the variability of extended *HLA-DRB1* haplotypes in relation to the other traditional HLA genes and a selected group of MHC class III genes. A total of 150 healthy Finnish individuals were included in the study. Subjects were genotyped for HLA alleles (*HLA-A, -B, -DRB1, -DQB1*, and *-DPB1*). The polymorphism of *TNF, LTA, C4, BTNL2* and *HLA-DRA* genes was studied with 74 SNPs (single nucleotide polymorphism). The *C4A* and *C4B* gene copy numbers and a 2-bp silencing insertion at exon 29 in *C4A* gene were analysed with quantitative genomic realtime-PCR. The allele frequencies for each locus were calculated and haplotypes were constructed using both the traditional HLA alleles and SNP blocks. The most frequent Finnish *A∼B∼DR* -haplotype, uncommon in elsewhere in Europe, was A*03∼B*35∼DRB1*01∶01. The second most common haplotype was a common European ancestral haplotype AH 8.1 (A*01∼B*08∼DRB1*03∶01). Extended haplotypes containing *HLA-B*, *TNF* block, *C4* and *HLA-DPB1* strongly increased the number of *HLA*-*DRB1* haplotypes showing variability in the extended *HLA-DRB1* haplotype structures. On the contrary, *BTNL2* block and *HLA-DQB1* were more conserved showing linkage with the *HLA-DRB1* alleles. We show that the use of *HLA-DRB1* haplotypes rather than single *HLA-DRB1* alleles is advantageous when studying the polymorphisms and LD patters of the MHC region. For disease association studies the *HLA-DRB1* haplotypes with various MHC markers allows us to cluster haplotypes with functionally important gene variants such as *C4* deficiency and cytokines *TNF* and *LTA*, and provides hypotheses for further assessment. Our study corroborates the importance of studying population-specific MHC haplotypes.

## Introduction

The Major Histocompatibility Complex located on chromosome 6p21 has a complex allelic structure with extended linkage disequilibrium (LD) and polymorphism. The traditional human leukocyte antigen (HLA) genes encode the cell-surface antigen-presenting proteins, the HLA molecules, and fall into two major MHC classes; Class I (*HLA-A, -C,* and *-B*) and Class II (*HLA-DRB1*, *-DQB1*, and *–DPB1*). Many non-HLA genes related to immune responses e.g. *tumor necrosis factor* (*TNF*), *lymphotoxin-alpha* (*LTA*), *complement C4* genes (*C4A* and *C4B*) and *butyrophilin-like protein 2* (*BTNL2*), are located in the MHC class III region that resides between the MHC Class I and II regions [1]–[3]. The location of recombination hotspots and the length of LD blocks (genomic fragments inherited together) are population specific [1], [4], [5]. Interestingly, it has been shown that the Finnish population has distinctive population substructure compared with other Europeans [6]–[8].

The HLA genes play a critical role in hematopoietic stem cell transplantation, and HLA mismatching has been associated with graft failure and graft-versus-host disease [9], [10]. In addition to the traditional HLA genes, it has been suggested that the HLA-SNP haplotypes influence on the outcome of the transplantation [11]. The association between MHC and diseases has been known for decades. The polymorphic *HLA-DRB1* has been associated with several complex and infectious diseases, such as acute coronary syndrome [12], HSV2 related meningitis [13], type I diabetes, celiac disease and multiple sclerosis (reviewed in [14]). Recently, genome wide association analyses (GWA) have increased the number of MHC gene associations with several drug-induced hypersensitivity reactions, autoimmune, infectious and inflammatory disorders [15]–[20] (GWAS Integrator, http://genome.ucsc.edu). However, the predisposing HLA-alleles are common in the healthy population. Other genetic and environmental triggers are required for disease susceptibility [15], [21]. Thus, the complexity of MHC makes the variant(s) responsible for the causal effect difficult to pinpoint. As the traditional HLA typing is considered rather expensive and laborious and the analysis of MHC regions is complex, many studies do not probe MHC associations more closely. Thus the functionality of the variants remains uncertain although identification of a specific risk HLA allele could help to understand a disease and its causality further.

At present, for transplantation and many diagnostics proposes, traditional HLA typing still remains as the most used method [14]. De Bakker [22] presented a tag-SNP based HLA-typing method as an alternative solution for traditional HLA typing. It has promoted the screening of certain disease related HLA markers [21], [23]. However, not only HLA allele and haplotype frequencies [8], [24] but also the SNP content of haplotypes differ in ethnically diverse populations complicating the imputation process. It is clear that more information on the precise content of HLA haplotypes is needed for transplantation, disease association, anthropological, and epidemiological studies [8], [25].

In this study we focused on studying *HLA-DRB1* haplotype structures in a Finnish population. The allelic diversities of other traditional HLA-genes and selected group of MHC class III genes were included and extended haplotypes inferred. We aimed to interpret the haplotype diversities in relation to *HLA-DRB1* locus because of its higher amount of polymorphism when compared to the other MHC Class II genes (IMGT database, [26]) and its well-known associations with several complex diseases. The selected MHC genes *TNF, LTA, C4, BTNL2,* and *HLA-DRA* all have been associated with several autoimmune or infectious diseases, as well. We hypothesized that the use of longer MHC blocks, rather than single alleles, could be advantageous when studying the polymorphisms in the MHC region.

## Materials and Methods

### Study Population

The study consists of one hundred and fifty (150) healthy Finnish individuals who were randomly selected. Description of the sample set and the DNA extraction has previously been published by Seppänen et al. [17]. Briefly, samples from 49 males and 101 females with mean age of 33.7 years (range 18–60), were used. The local ethical committee (The Ethics Committee: Department of Medicine, Hospital District of Helsinki and Uusimaa) approved the study protocol (Dnro 6/E5/2001,25.1.2001). All the contributors provided the written informed consent.

### Genotyping of HLA Genes

The HLA genotyping and/or analysis was carried out in an EFI (European Federation for Immunogenetics) accredited HLA Laboratory. The genotyping of *HLA-A, -B* and *–DPB1* genes were performed using sequence specific primers (SSP: Olerup SSP AB, Stockholm, Sweden). The *HLA-DRB1* alleles were detected using sequence based typing (SBT; InvitrogenTM, Life Technologies, Carlsbad, CA, USA). *HLA-DQB1* alleles were detected with a panel of lanthanide-labeled oligonucleotide probes [27]. The reactions were performed according to manufacturers’ instructions giving at least four-digit resolution (for example, HLA-DRB1*01∶01) for *HLA-DRB1* and *–DPB1*, and at least two-digit resolution (for example, HLA-A*02) for *HLA-A, -B* and *-DQB1*. For resolving ambiguities, high resolution SSPs or SBT HARPs (heterozygous ambiguity resolving primers) were used. The HLA alleles were assessed using HLA nomenclature release 3.5.0 (IMGT/HLA database) and carefully interpreted by two persons.

### SNP Selection and Genotyping

We aimed to genotyped *LTA, TNF, BTNL2* and *HLA-DRA* genes with SNPs. All the selected genes were covered entirely including 5′ and 3′- flanking regions. SNPs were chosen from the HapMap database [28] and/or from the public dbSNP database (http://www.ncbi.nlm.nih.gov/projects/SNP). Information about the validation status, tagging quality, minor allele frequency (>0.01) and gene structure were used for selecting the SNPs. Altogether seventy-four SNPs were chosen from the two gene regions, *TNF∼LTA,* (here referred as *TNF* block) and *BTNL2∼HLA-DRA* (here referred as *BTNL2* block).

SNP genotyping was performed using the Sequenom MassArray iPLEX system (Sequenom, San Diego, CA, USA). In the iPLEX assay, the SNP alleles are separated based on the differences of the single base extension (SBE) products. Manufacture’s instructions were used to design the assays (AssayDesign software) and to perform the multiplex PCR and the iPLEX reaction using 9–10 ng of DNA as a template.

The complement *C4A* and *C4B* gene copy numbers and a 2-bp silencing insertion at exon 29 (CT) in *C4A* gene were analysed by quantitative genomic realtime-PCR Rotor-Gene 6000 (Corbett Research, Sydney, Australia) according to Paakkanen et al. [29]. The C4 allotypes were determined by immunofixation [17]. One subject was excluded from the analysis.

### Statistical Analysis

Allele, phenotype and haplotype frequencies were calculated by direct counting. To detect significant departure from Hardy-Weinberg equilibrium (p<0.001), Haploview (SNPs) [30] or ARLEQUIN 3.11 (HLA genes) [31], were used.

SNP haplotypes were constructed using Haploview [30]. Multi-locus haplotype frequencies and recombination rates were estimated from allele data using the Bayesian method with PHASE v. 2.1.1 [32]. The haplotypes were constructed using all the selected markers simultaneously. Taken into account the small sample size and to exclude unreliable haplotypes, only haplotypes greater than 1% (observed more than 3 times) were used in the analysis [24], [33]–[35].

The LD measures (D′ and r^2^) were determined using either the Haploview software (biallelic markers) [30] or the ARLEQUIN 3.11 software (multiallelic markers) [31]. At this step, only one SNP from a LD block was chosen for further analysis. It has been shown that for multiallelic loci, D′ estimates the strength of LD (>0.80 strong LD, −0.5 moderate LD, −0 weak LD) better than r^2^ [36]. The R-package ‘ape’ was used to perform a Neighbor-joining algorithm according to the method of Saitou and Nei [37]. The HapMap data was used for illustrating the recombination hotspots in the MHC region [28]. The proxy SNPs (r^2^>0.9) for genotyped SNPs were detected using software SNAP [38].

## Results

### Genotyping of HLA-alleles

The HLA alleles and their frequencies are given in parallel with the phenotype frequency in Table 1. HLA allele distributions followed the Hardy–Weinberg equation. A total of 91 HLA alleles (15, 21, 26, 11 and 18 alleles in *HLA-A, -B, -DRB1, -DQB1* and -*DPB1*, respectively) were noted. In the Finnish population, two *HLA-A* alleles accounted for >66% and five *HLA-DPB1* alleles for >91% of the variation at the loci. Allele frequencies of other HLA loci were more equally distributed. These observations are consistent with the previous Finnish studies [39], [40].

**Table 1:** The Finnish HLA allele and phenotype frequencies.

HLA-A	Allele		Phenotype		HLA-DRB1	Allele		Phenotype		HLA-DQB1	Allele		Phenotype		HLA-DPB1	Allele		Phenotype	
Allele	n	f	n	f	Allele	n	f	n	f	Allele	n	f	n	f	Allele	n	f	n	f
*02	126	0.420	101	0.673	*15∶01	44	0.147	42	0.280	*06∶02	44	0.147	42	0.280	*04∶01	103	0.343	84	0.553
*03	71	0.237	61	0.407	*01∶01	44	0.147	38	0.253	*05∶01	48	0.160	40	0.267	*04∶02	61	0.203	55	0.367
*01	23	0.077	23	0.153	*08∶01	37	0.123	33	0.220	*02^#^	41	0.137	39	0.260	*02∶01	48	0.160	43	0.287
*24	20	0.067	20	0.133	*03∶01	29	0.097	28	0.187	*03∶01	45	0.150	39	0.260	*03∶01	43	0.143	39	0.260
*68	14	0.047	14	0.093	*13∶01	27	0.090	26	0.173	*04^#^	38	0.127	34	0.227	*01∶01	17	0.057	17	0.113
*11	12	0.040	12	0.080	*04∶01	21	0.070	21	0.140	*03∶02	27	0.090	27	0.180	*05∶01	10	0.033	10	0.067
*32	10	0.033	10	0.067	*07∶01	16	0.053	15	0.100	*06∶03	27	0.090	26	0.173	*19∶01	3	0.010	3	0.020
*31	7	0.023	7	0.047	*13∶02	13	0.043	12	0.080	*03∶03	13	0.043	12	0.080	*13∶01	2	0.007	2	0.013
*29	5	0.017	5	0.033	*11∶01	12	0.040	11	0.073	*06∶04	13	0.043	12	0.080	*14∶01	2	0.007	2	0.013
*26	4	0.013	4	0.027	*12∶01	11	0.037	11	0.073	*05∶02	2	0.007	2	0.013	*17∶01	2	0.007	2	0.013
*30	3	0.010	3	0.020	*09∶01	10	0.033	9	0.060	*05∶03	2	0.007	2	0.013	*20∶01	2	0.007	2	0.013
*66	2	0.007	2	0.013	*04∶04	6	0.020	6	0.040	**11 alleles**					*06∶01	1	0.003	1	0.007
*23	1	0.003	1	0.007	*04∶08	6	0.020	6	0.040						*104∶01	1	0.003	1	0.007
*25	1	0.003	1	0.007	*04∶03	4	0.013	4	0.027						*15∶01	1	0.003	1	0.007
*33	1	0.003	1	0.007	*14∶01:01G	3	0.010	3	0.020						*16∶01	1	0.003	1	0.007
**15 alleles**					*04∶02	2	0.007	2	0.013						*24∶01	1	0.003	1	0.007
					*08∶03	2	0.007	2	0.013						*81∶01	1	0.003	1	0.007
**HLA-B**	**Allele**		**Phenotype**		*10∶01	2	0.007	2	0.013						*85∶01	1	0.003	1	0.007
**Allele**	**n**	**f**	**n**	**f**	*11∶04	2	0.007	2	0.013						**18 alleles**				
*15	43	0.143	41	0.273	*14∶02	2	0.007	2	0.013										
*07	40	0.133	40	0.267	*16∶01	2	0.007	2	0.013										
*35	38	0.127	35	0.233	*01∶03	1	0.003	1	0.007										
*08	30	0.100	29	0.193	*04∶07	1	0.003	1	0.007										
*27	27	0.090	26	0.173	*08∶02	1	0.003	1	0.007										
*44	26	0.087	26	0.173	*08∶04	1	0.003	1	0.007										
*40	26	0.087	24	0.160	*11∶03	1	0.003	1	0.007										
*51	21	0.070	20	0.133	**26 alleles**														
*39	11	0.037	11	0.073															
*13	7	0.023	7	0.047															
*56	6	0.020	6	0.040															
*18	5	0.017	5	0.033															
*47	4	0.013	4	0.027															
*57	4	0.013	4	0.027															
*41	3	0.010	3	0.020															
*38	2	0.007	2	0.013															
*53	2	0.007	2	0.013															
*55	2	0.007	2	0.013															
*14	1	0.003	1	0.007															
*45	1	0.003	1	0.007															
*50	1	0.003	1	0.007															
**21 alleles**																			

n = number of alleles/phenotypes.

Number of subjects = 150; Number of alleles = 300.

The phenotype or carrier positivity contains one or two copies of the variant.

HLA-DRB1*14∶01:01G stands for DRB1*14∶01:01 and DRB1*14∶54.

# 2-digit resolution genotyping for HLA-DQB1*02 and *04.

### HLA Haplotypes

Three-locus haplotypes between *HLA-DRB1* and MHC Class I (*HLA-A* and *–B*; Table 2) and MHC Class II (*HLA-DQB*1 and *-DPB1*; Table 3) were constructed. Haplotypes having frequencies higher than 1.0% are presented in Table 2 and 3 and compared with different populations that had reported the *A∼B∼DRB1* and *DQB1∼-DPB1∼DRB1* haplotypes in The Allele Frequency Net Database [41]–[46]. The three most common *A∼B∼DRB1* haplotypes in the Finnish population were A*03∼B*35∼DRB1*01∶01 (7.1%), A*01∼B*08∼DRB1*03∶01 (4.0%) and A*02∼B*07∼DRB1*15∶01 (3.5%). The second most common HLA-DRB1*01∶01 haplotype had HLA-B*07 instead of HLA-B*35. The two-locus haplotypes (*DRB1∼DPB1*, *DQB1∼DPB1* and *B∼DRB1*) are presented in Table S1.The linkage between *HLA-DRB1* and *HLA-DQB1* was stronger than between *HLA-DRB1* and *HLA-DPB1* (Table S2).

**Table 2:** The Finnish *A∼B∼DRB1* haplotype frequencies (>1%) and comparison with other populations [41]–[46].

Haplotype A∼B∼DRB1			Finland (n = 150)	Sami (n = 130) [44]	Russia (n = 207) [46]	Ireland (n = 1000) [41]–[43]
HLA-A	HLA-B	HLA-DRB1	f	f	f	f
*01	*08	*03∶01	0.040	0.039	0.050	0.09
*02	*07	*15∶01	0.035	0.038	–	0.03
*02	*27	*08∶01	0.027	0.014	–	–
*02	*15	*13∶01	0.023	0.031	0.012	–
*02	*15	*04∶01	0.019	0.023	–	–
*02	*15	*08∶01	0.019	–	–	–
*02	*13	*07∶01	0.016	–	0.021	–
*02	*15	*15∶01	0.013	–	–	–
*02	*44	*04∶01	0.012	–	0.019	0.04
*02	*40	*13∶02	0.011	–	–	–
*03	*35	*01∶01	0.071	0.031	0.033	–
*03	*08	*03∶01	0.021	–	–	–
*03	*15	*08∶01	0.017	0.015	–	–
*03	*07	*01∶01	0.014	–	0.019	–
*03	*07	*13∶01	0.012	–	–	–
*03	*07	*15∶01	0.011	0.042	0.047	0.05
*11	*07	*15∶01	0.015	–	–	–

f = frequency.

**Table 3:** The Finnish *DRB1*∼*DQB1∼DPB1* haplotype frequencies (>1%) and comparison with other populations [41]–[46].

*Haplotype DRB1∼DQB1∼DPB1*			Finland (n = 150)	Ireland (n = 250) [41]–[43]	Greece (n = 246) [45]
HLA-DRB1	HLA-DQB1	HLA-DPB1	f	f	f
*01∶01	*05∶01	*04∶02	0.059	0.024	–
*01∶01	*05∶01	*04∶01	0.043	0.028	0.035
*01∶01	*05∶01	*02∶01	0.040	0.011	–
*03∶01	*02	*01∶01	0.049	0.021	0.014
*03∶01	*02	*04∶01	0.020	0.098	0.015
*04∶01	*03∶02	*04∶01	0.026	0.013	–
*04∶01	*03∶02	*04∶02	0.016	–	–
*04∶04	*03∶02	*02∶01	0.013	–	–
*04∶08	*03∶01	*02∶01	0.016	–	–
*07∶01	*02	*04∶01	0.023	0.017	0.014
*08∶01	*04	*03∶01	0.071	–	–
*08∶01	*04	*04∶01	0.033	–	–
*08∶01	*04	*04∶02	0.014	–	–
*09∶01	*03∶03	*04∶02	0.022	–	–
*11∶01	*03∶01	*04∶02	0.013	–	–
*11∶01	*03∶01	*04∶01	0.012	–	0.036
*11∶01	*03∶01	*02∶01	0.012	–	0.023
*12∶01	*03∶01	*04∶01	0.025	–	–
*13∶01	*06∶03	*04∶01	0.027	0.014	0.016
*13∶01	*06∶03	*02∶01	0.023	–	0.023
*13∶01	*06∶03	*05∶01	0.012	–	–
*13∶02	*06∶04	*03∶01	0.030	–	–
*13∶02	*06∶04	*04∶02	0.013	–	–
*15∶01	*06∶02	*04∶01	0.090	0.149	0.015
*15∶01	*06∶02	*04∶02	0.020	–	–
*15∶01	*06∶02	*05∶01	0.014	–	–

f = frequency.

Total number of haplotypes in the population sample (Haplotypes/2n) shown in frequencies (f).

### SNP Analysis

Altogether 74 SNPs were successfully genotyped. The average success rate was 99% and no discrepancies were observed. Fourteen SNPs were excluded due to minor allele frequency (<0.01) or HWE (<0.001) and five SNPs were excluded as they were in total LD (r^2^ = 1) with another SNP. There can be found several proxy SNPs (SNPs in strong LD) for our SNPs (examples shown in Table S3) [38]. Many of the proxy SNPs have been previously associated with diseases (http://www.snp-nexus.org/).

A summary of the accepted SNPs (n = 55) is given in Table S4. The allele frequencies of our genotyped SNPs did not differ significantly from the HapMap (CEU population, European decent) [28], except the twelve SNPs in *BTNL2* or *HLA-DRA* genes (Table S4). The LD structure of the SNPs (*TNF* or *BTNL2* block) is presented in the Figure S1. As expected, due to the SNP selection criteria the pairwise LD between SNPs was always r^2^<1.

**Figure 1:**
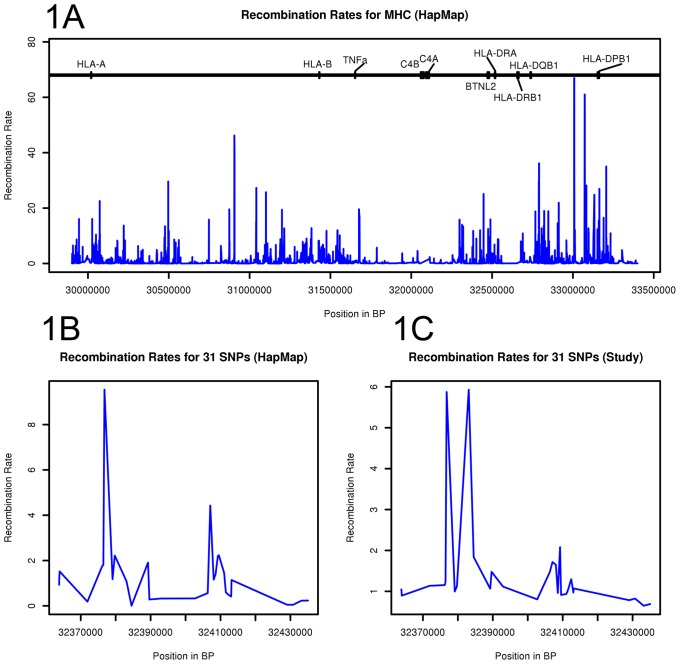
Recombination rates in MHC region. 1A: The recombination rates for (HapMap [28]) in MHC region. 1B: Recombination rates in HapMap [28] for the 31 SNPs that are common in the current study and HapMap. 1C: Recombination rates for the 31 SNPs in the study (that are common in the study and HapMap [28])). The highest recombination rate was observed in the *BTNL2* promoter region.

Previously using HapMap data [28], high recombination rates have been observed between *HLA-A* and *–B* loci, upstream and downstream of *TNF*, in the *BTNL2* region and between *HLA-DQB1* and –*DPB1* loci (Figure 1A). Here, the recombination rate estimation was performed using the HLA alleles (*HLA-A, -B, -DRB1, -DQB1, -DPB1*), C4 gene copy numbers and SNPs (n = 55). Thirty-one SNPs were common in both HapMap and in our study. The location of the highest recombination rate in the Finnish sample was observed in the *BTNL2* promoter region corresponding to HapMap data (Figures 1 B and C).

### Haplotypes of *TNF, BTNL2* and *C4*

SNP haplotypes were constructed. The rare haplotypes (observed less than 3 times i.e. frequency <1%) were excluded at this point. Nine 13-SNP haplotypes of *TNF* block (Table 4) and twelve 42-SNP haplotype of *BTNL2* block (Table 6) were observed with frequencies ranging from 3% to 21% and 1.3% to 15%, respectively. In both regions, there seemed to be four to five common haplotypes of almost equal frequency in addition to the less common haplotypes. *C4A* and *C4B* gene copy numbers were constructed into haplotypes and the results are shown in Table 8. As opposed to *TNF* and *BTNL2* haplotypes, there was one haplotype, which covered more than half of the observed haplotypes (*C4_1* haplotype, 59%). The frequency of the next common haplotype was less than 20%, showing a sharp decrease.

**Table 4:** *TNF* blocks (*TNF_1– TNF_9*) and their frequencies (>1.0%).*HLA-DRB1* Haplotypes with *TNF, BTNL2* and *C4* Blocks.

SNP	*TNF_1*	*TNF_2*	*TNF_3*	*TNF_4*	*TNF_5*	*TNF_6*	*TNF_7*	*TNF_8*	*TNF_9*	
rs2009658	C	C	C	C	G	C	C	G	C	
rs2239704	A	C	C	C	C	A	A	C	C	
rs2229094	T	T	T	C	C	T	T	C	T	
rs2229092	A	A	A	A	A	A	A	C	A	
rs1041981	C	A	A	C	C	C	C	C	A	
rs1799964	T	T	T	T	C	T	T	C	T	
rs1799724	C	C	C	C	C	C	T	C	C	
rs1800629	G	G	A	G	G	G	G	G	G	
rs361525	G	G	G	G	G	G	G	G	G	
rs3093664	A	A	A	A	A	A	A	A	G	
rs769178	G	G	G	G	G	G	T	G	G	
rs3093553	T	T	T	T	T	T	T	T	T	
rs2256965	A	G	G	A	G	G	A	G	G	
F	0.210	0.167	0.153	0.130	0.090	0.080	0.057	0.047	0.030	0.96#

**Table 5:** Two-locus haplotypes of *HLA-DRB1* with *TNF* block (>1.0%)#.

DRB1 allele	TNF block	f
*01∶01	TNF_4	0.103
*03∶01	TNF_3	0.076
*04∶01	TNF_2	0.022
*04∶01	TNF_5	0.027
*04∶08	TNF_1	0.016
*07∶01	TNF_1	0.040
*08∶01	TNF_1	0.027
*08∶01	TNF_2	0.048
*08∶01	TNF_6	0.030
*09∶01	TNF_6	0.025
*11∶01	TNF_3	0.014
*13∶01	TNF_1	0.017
*13∶01	TNF_2	0.035
*13∶02	TNF_9	0.026
*15∶01	TNF_1	0.075
*15∶01	TNF_4	0.016
*15∶01	TNF_7	0.014

f = frequency.

# Rare haplotypes were excluded from the analysis.

**Table 6:** *BTNL2* blocks (*BTNL2_1– BTNL2_12*) and their frequencies (>1.0%).

SNP	*BTNL2_1*	*BTNL2_2*	*BTNL2_3*	*BTNL2_4*	*BTNL2_5*	*BTNL2_6*	*BTNL2_7*	*BTNL2_8*	*BTNL2_9*	*BTNL2_10*	*BTNL2_11*	*BTNL2_12*
rs28362678	T	C	C	C	C	T	C	C	C	C	C	C
rs2076530	C	T	C	T	T	C	T	T	T	T	T	T
rs9268480	C	C	T	C	C	C	C	C	C	C	C	C
rs2076529	C	T	C	T	T	C	T	T	T	T	T	T
rs3793127	C	C	T	C	C	C	C	C	C	C	C	C
rs28362683	G	G	G	A	G	A	G	G	G	G	G	G
rs3763311	C	C	T	C	C	C	C	C	C	C	C	C
rs3763312	G	G	A	G	G	G	G	G	G	G	G	G
rs3763313	C	A	A	C	A	A	A	A	A	A	A	C
rs3763317	T	C	T	T	C	C	C	C	C	C	C	T
rs5007259	C	T	C	C	T	T	T	T	T	T	T	C
rs17208888	G	G	G	G	G	G	G	A	G	G	G	G
rs9405098	G	G	G	G	G	G	G	G	G	G	G	G
rs9268528	A	A	G	A	A	A	A	G	A	G	G	A
rs9268541	T	T	T	T	T	T	T	T	T	C	C	T
rs2395166	C	C	T	T	T	T	T	T	T	T	T	C
rs3135365	T	G	T	T	T	T	T	T	T	T	T	T
rs3135363	T	T	T	T	C	C	C	C	C	T	T	T
rs3135351	G	G	G	G	T	T	T	G	T	G	G	G
rs3135344	G	G	A	A	A	A	A	A	A	A	A	G
rs3129843	A	A	A	A	G	A	A	A	A	A	A	A
rs3135341	T	G	T	T	T	T	T	T	T	T	T	G
rs2027856	C	C	C	T	C	C	C	C	C	C	C	C
rs3129871	C	A	C	A	C	A	C	C	C	A	A	A
rs9405035	G	G	G	G	G	G	G	G	G	G	G	G
rs9268644	A	A	C	C	A	A	A	C	A	A	C	C
rs3129877	A	G	G	G	A	A	A	G	A	G	G	G
rs3135392	T	G	G	T	T	T	T	G	T	T	T	T
rs3129882	A	G	A	G	A	A	A	G	A	G	G	G
rs8084	C	A	C	A	A	A	A	A	A	A	A	A
rs2239804	A	A	G	A	A	A	A	G	A	A	A	A
rs11544315	G	G	G	G	G	G	G	G	G	G	G	A
rs3177928	A	G	G	G	G	G	G	G	G	G	G	A
rs3135388	C	T	C	C	C	C	C	C	C	C	C	C
rs2213585	T	C	T	C	C	C	C	T	C	C	C	T
rs6937545	C	A	C	C	A	A	A	C	A	C	C	C
rs9268833	C	C	T	C	C	C	C	C	C	C	C	C
rs6919855	T	C	T	T	T	T	T	C	T	T	T	T
rs7766843	C	C	C	T	T	T	T	C	T	T	T	C
rs2395185	G	G	T	G	G	G	G	G	G	G	G	T
rs9268979	T	T	C	C	C	C	C	T	C	C	C	C
rs7748472	A	A	A	A	A	A	G	A	A	A	A	A
f	0.150	0.143	0.137	0.103	0.097	0.053	0.040	0.040	0.020	0.020	0.017	0.013

**Table 7:** Two-locus haplotypes of *HLA-DRB1* with *BTNL2* block (>1.0%)#.

HLA-DRB1	BTNL2 block	f
*01∶01	BTNL2_1	0.147
*03∶01	BTNL2_5	0.097
*04∶01	BTNL2_3	0.064
*04∶03	BTNL2_3	0.013
*04∶08	BTNL2_3	0.020
*07∶01	BTNL2_3	0.023
*07∶01	BTNL2_12	0.013
*08∶01	BTNL2_4	0.103
*11∶01	BTNL2_8	0.037
*12∶01	BTNL2_10	0.020
*12∶01	BTNL2_11	0.016
*13∶01	BTNL2_6	0.053
*13∶02	BTNL2_7	0.040
*15∶01	BTNL2_2	0.143

#Rare haplotypes were excluded.

f = frequency.

**Table 8:** C4 blocks (C4_1– C4_6) and their frequencies (>1.0%).

C4 gene	*C4_1*	*C4_2*	*C4_3*	*C4_4*	*C4_5*	*C4_6*
C4A	1	2	1	Q0	InsCT	2
C4B	1	Q0	Q0	1	1	1
f	0.59	0.17	0.09	0.08	0.03	0.03

The significant pair-wise LD [(D′>0.80, r^2^>0.35, P value (P) <0.05)] between *HLA-DRB1* alleles and other markers is presented in Table 10. The *HLA-DRB1* allele distribution (%) presented in Table S2 shows how *HLA-DRB1* alleles are clustered with MHC class I, II and III alleles and MHC blocks.

**Table 9:** Two-locus haplotypes of HLA-DRB1 with C4 block (>1.0%)#.

HLA-DRB1	C4 block	f
*01∶01	2	0.083
*01∶01	1	0.032
*01∶01	3	0.029
*03∶01	4	0.068
*03∶01	1	0.022
*04∶01	1	0.038
*04∶01	2	0.026
*04∶04	1	0.019
*04∶08	1	0.019
*07∶01	1	0.046
*08∶01	1	0.082
*08∶01	3	0.041
*09∶01	1	0.029
*11∶01	1	0.036
*12∶01	1	0.024
*13∶01	1	0.045
*13∶01	2	0.032
*13∶02	5	0.030
*15∶01	1	0.120
*15∶01	6	0.016

# Rare haplotypes were excluded.

Q0 = null allele.

InsCT = insertion in C4A gene.

f = frequency.

**Table 10:** *HLA-DRB1* alleles in strong LD# with *TNF, C4* and *BTNL2* blocks and *HLA-DQB1* and *-DPB1* alleles. LD is measured with D′/r^2^.

	HLA-DRB1*01∶01	HLA-DRB1*03∶01	HLA-DRB1*04∶01	HLA-DRB1*08∶01	HLA-DRB1*09∶01	HLA-DRB1*11∶01	HLA-DRB1*12∶01	HLA-DRB1*13∶01	HLA-DRB1*13∶02	HLA-DRB1*15∶01
*TNF_3*	–	0.80/0.37	–	–	–	–	–	–	–	–
*TNF_9*	–	–	–	–	–	–	–	–	0.88/0.53	–
*C4_4*	–	0.73/0.46	–	–	–	–	–	–	–	–
*C4_5*	–	–	–	–	–	–	–	–	0.90/0.61	–
*BTNL2-1*	1.0/0.97	–	–	–	–	–	–	–	–	–
*BTNL2-2*	–	–	–	–	–	–	–	–	–	1.0/0.97
*BTNL2-3*	–	–	0.95/0.43	–	–	–	–	–	–	–
*BTNL2-4*	–	–	–	1.0/0.82	–	–	–	–	–	–
*BTNL2-5*	–	1.0/1.0	–	–	–	–	–	–	–	–
*BTNL2-6*	–	–	–	–	–	–	–	1.0/0.57	–	–
*BTNL2-7*	–	–	–	–	–	–	–	–	1.0/0.92	–
*BTNL2-8*	–	–	–	–	–	0.91/0.84	–	–	–	–
*BTNL2-10*	–	–	–	–	–	–	1.0/0.54	–	–	–
*BTNL2-11*	–	–	–	–	–	–	1.0/0.45	–	–	–
DQB1*02	–	1.0/0.68	–	–	–	–	–	–	–	–
DQB1*03∶03	–	–	–	–	1.0/0.76	–	–	–	–	–
DQB1*04	–	–	–	1.0/0.97	–	–	–	–	–	–
DQB1*05	1.0/0.90	–	–	–	–	–	–	–	–	–
DQB1*06∶02	–	–	–	–	–	–	–	–	–	1.0/1.0
DQB1*06∶03	–	–	–	–	–	–	–	1.0/1.0	–	–
DQB1*06∶04	–	–	–	–	–	–	–	–	1.0/1.0	–
DPB1*01∶01	–	0.87/0.43	–	–	–	–	–	–	–	–

# Here the strong LD (D′>0.80) presented if r^2^>0.35 and P value <0.05.

The linkage between *HLA-DRB1* and *TNF* block (Table 5) indicates that a given *HLA-DRB1* allele was combined with different *TNF* blocks. Two cases, HLA-DRB1*03∶01 and *TNF_3* and HLA-DRB1*13∶02 and *TNF_9* showed strong LD (D′ = 0.80; D′ = 0.88, respectively, Table 10).

Contrary to *TNF* block, the linkage between *HLA-DRB1* and *BTNL2* block (Table 7) showed that mostly *HLA-DRB1* alleles (*01∶01, *03∶01, *04∶01, *08∶01, *11∶01, *13∶02 and *15∶01) were strongly linked with *BTNL2* blocks (Table 10, Table S2). However, there were some exceptions such as HLA-DRB1 *12∶01 and *13∶01 (Table S2).

The *DRB1∼C4* haplotypes behaved like the *DRB1∼TNF* haplotypes showing association with different *HLA*-*DRB1* alleles (Table 9). *C4B* null allele (haplotypes *C4_2* and *C4_3*) were found with DRB1*01∶01, *04∶01, *08∶01 and *13∶01. HLA-DRB1*03∶01 had C4AQ0 (C4_4; D′ = 0.73; Table 10), but also a haplotype without a *C4A* null allele (*C4_1*) was observed. HLA-DRB1*13∶02 had typically an insertion in the *C4A* gene (*C4_5*; D´ = 0.90 Table 10).

To summarize the LD and haplotype analysis and to the polymorphism of the MHC region and its relation to *HLA-DRB1*, an additional four-locus haplotype (*HLA-DRB1*, *TNF* block, *BTNL2* block and *C4;* Table 11) and a six-locus haplotype (*HLA-DRB1*, *HLA-B*, *TNF* block, *BTNL2* block, *C4A* and *C4B* allotypes; Table S5) were performed. The results showed that the extended *HLA-DRB1* haplotypes were broken down when *HLA-B,* C4 allotypes and *TNF* block were taken into account.

**Table 11:** The extended *HLA-DRB1* haplotypes with *TNF*, *C4* and *BTNL2* blocks (>1%).

HLA-DRB1	TNF block	C4 block	BTNL2 block	f
*01∶01	TNF_4	2	BTNL2_1	0.059
*01∶01	TNF_4	3	BTNL2_1	0.026
*01∶01	TNF_4	1	BTNL2_1	0.017
*01∶01	TNF_8	2	BTNL2_1	0.012
*03∶01	TNF_3	4	BTNL2_5	0.065
*03∶01	TNF_3	1	BTNL2_5	0.011
*04∶01	TNF_5	1	BTNL2_3	0.024
*04∶01	TNF_2	2	BTNL2_3	0.022
*04∶08	TNF_1	1	BTNL2_3	0.013
*07∶01	TNF_1	1	BTNL2_3	0.023
*08∶01	TNF_6	1	BTNL2_4	0.028
*08∶01	TNF_2	1	BTNL2_4	0.022
*08∶01	TNF_2	3	BTNL2_4	0.022
*08∶01	TNF_1	1	BTNL2_4	0.016
*11∶01	TNF_5	1	BTNL2_8	0.011
*13∶01	TNF_2	2	BTNL2_6	0.014
*13∶01	TNF_1	1	BTNL2_6	0.012
*13∶02	TNF_9	5	BTNL2_7	0.027
*15∶01	TNF_1	1	BTNL2_2	0.059
*15∶01	TNF_7	1	BTNL2_2	0.016
*15∶01	TNF_5	1	BTNL2_2	0.012

f = haplotype frequency.

To further analyze the structure of *TNF* and *BTNL2* blocks we created phylogenetic trees of the genetic distance using sequence similarities (Figure S2). Three branches of *TNF* were observed, from which two branches, *TNF_3* and *_9* formed haplotypes with HLA-DRB1*03∶01 and HLA-DRB1*13∶02, respectively, which we previously showed with high LD. The third branch divided into three groups, first with *TNF_2*, second with *TNF_4, _5* and *_8* and third with *TNF_1, _6*, and *_7*. *C4BQ0* related *TNF* haplotypes, *TNF_2* and *TNF_4*, belonged to the same main branch. The phylogenetic tree of *BTNL2* blocks showed that *BTNL2_5* and _*7* (in linkage with HLA-DRB1*03∶01 and *13∶02, respectively*) and BTNL2_6 (*in linkage with HLA-*13∶01) had different BTNL2 block structure than the rest. HLA-DRB1*12∶01 alleles had either BTNL2 blocks BTNL2_10 or _11. HLA-DRB1*11∶01 and *15∶01 had structurally similar BTNL2 blocks, BTNL2_8 and _2, respectively. HLA-DRB1*01∶01, HLA-DRB1*04 alleles and HLA-DRB1*07∶01 and *08∶01 formed a wide branch with similar BTNL2 blocks *_3, _4, and _8*, *_10 and _11* (in linkage with HLA-DRB1*01∶01, *04∶01-08, *07∶01, *08∶01, *11∶01, *12∶01, *15∶01) and *BTNL2_9* (in linkage with HLA-DRB1*13∶01).

## Discussion

To our knowledge, no extensive study exist that combines information from *HLA-A,-B,-DRB1,-DQB1* and *–DPB1* alleles with *TNF*, *BTNL2* and complement *C4* blocks. In this study, we addressed (i) the diversity of extended *HLA-DRB1* haplotypes covering the MHC class I, II and III regions, (ii) the shared MHC or SNP markers in extended *HLA-DRB1* haplotypes, and (iii) the challenge in detecting causal variants in the HLA data.

The most common Finnish *A∼B∼DR* –haplotype was A*03∼B*35∼DRB1*01∶01. Also other HLA-DRB1*01∶01 haplotypes with variable levels of LD were observed. According to The Allele Frequency Net Database [41], only in a few populations e.g. the Swedish Sami [44] and Russia [46] the A*03∼B*35∼DRB1*01∶01 was found with the frequency >2%. In Finland, the second most common haplotype was A*01∼B*08∼DRB1*03∶01 in high LD. This haplotype is not so common in Finns as in other Europeans and has been previously referred as the ancestral haplotype AH 8.1 or autoimmune haplotype [47], [48]. Another conserved haplotype with strong LD, but rare in the Finnish population, was HLA-DRB1*13∶02 reaching from *TNF* to *HLA-DQB1*.

The enrichment or loss of certain HLA haplotypes (Table 2) reflects the characteristics of the Finnish population structure, which has evolved through multiple genetic bottlenecks [6], [7]. Detailed information of population substructures was presented by the 16th International HLA and Immunogenic Workshop IHIW project (“Analysis of HLA Population Data” [8]. Most importantly, the multidimensional scaling (MDS) of *HLA-DRB1* revealed that the Finns and the Sami are closer to the North-East Asians than to other European populations [8]. In general, the HLA variation in Europe follows the North to Southeast axis corresponding to the previous principal component analysis (PCA) based results that utilized genome-wide SNP data [7]. Interestingly in Finland, the prevalence of certain HLA alleles have shown regional differences [40] e.g. HLA-B*35 being highest in the Eastern parts of Finland. Also GWASs have shown similar trends [7]. The HLA haplotype deviations in relation to the Finnish population substructure warrant replication studies. The population stratification is important e.g. for control selection [34].

We found that the majority of *HLA-DRB1* alleles were inherited as extended blocks from *BTNL2* to *HLA-DQB1*. In spite of the observed recombination rate in the *BTNL2* promoter region, most *DRB1∼BTNL2* blocks appeared to be conserved. Including *HLA-B*, *TNF, C4* and *HLA-DPB1* the number of extended *HLA*-*DRB1* haplotypes strongly increased. The positions of the MHC recombination sites vary between populations [49] explaining partly the non-replication of disease associations between populations (e.g. [19], [50], [51]).

Our multi-locus haplotype analysis shows that the extended *HLA-DRB1* haplotypes can be grouped according to functional similarities. Especially interesting for disease association studies are the *HLA-DRB1* haplotypes not common in the general population. Furthermore, *TNF* and *LTA* genes are cytokines involved in the activation of inflammatory processes; hence the *HLA-DRB1* haplotypes with gene expression related SNPs (rs2239704, rs1041981 and rs1800629) [2], [52] are plausible candidates for inflammatory diseases. For example, a rare HLA-DRB1*15∶01∼TNF_4 haplotype has a different nucleotide in rs2239704 compared with more frequent HLA-DRB1*15∶01∼TNF haplotypes. Interestingly, the same *TNF_4* is also found with HLA-DRB1*01∶01 shown to be associated with inflammatory reactions [53]. The extended *HLA-DRB1* haplotypes of HLA-DRB1*03∶01, *08∶01, *11∶01, *12∶01, *13∶02 and *15∶01 can be grouped according to exonic missense SNP (rs2076530) causing truncated protein of the T-cell inhibitor *BTNL2* [19]. Of the complement C4 proteins, the *C4A* null alleles were primarily found with two conserved haplotypes, HLA-DRB1*03∶01 (AH 8.1) and HLA-DRB1*13∶02. *C4B* null alleles were characteristically inherited with the most common Finnish haplotype A*03∼B*35∼DRB1*01∶01 or with HLA-DRB1*04∶01, *08∶01 and *13∶01. C4 null alleles have shown to be related to many diseases [18]. Taken together, the extended *HLA-DRB1* haplotype analysis can reveal predisposing/protective associations between markers and disease loci not detectable with a single MHC allele [34].

Overall, analysis of immunogenomic data is challenging. Resolving HLA allele ambiguity, phasing and LD calculation warrant particular expertise, and the traditional software tools (e.g. Haploview and PLINK) are not suitable for multiple loci polymorphic data like HLA [34], [54]. Haplotypes rather than single markers were used to decrease phasing errors [55]. Due to the strong LD, multiple SNPs may have corresponding statistical proof of association making the search for possible causal variants exceptionally difficult. To clarify the complexity, the known tag-SNP for HLA-DRB1*15∶01 (rs3135388) (see Figure S3), was shown to have at least 20 proxy SNPs (r^2^>0.9) with variable function and in different gene regions [14], [22], [38]. The SNPs’ allele frequency might be also population specific (rs2213585; Table S4), and thus the tag-SNPing (i.e. imputation) should not be used unless the ethnic background is known [56]. Here in this material, except the tag-SNP for HLA-DRB1*15∶01 (rs3135388) [14], [22], we did not detect any single tag-SNP for a specific *HLA-DRB1* allele. Indeed, large population specific cohorts and dense SNP genotyping is needed for detecting HLA tag-SNPs.

We acknowledge that the multiple ambiguous alleles, limited sample size, and rare HLA alleles can influence the haplotype phasing and LD leading to false positive results [34]. In case of small sample size, the study of the rare MHC haplotypes is challenging. Thus, we presented only frequent MHC haplotypes (>1%) and interpreted the LD between markers carefully [34], [35], [54], [55], [57]. The HLA allele distributions were consistent with the previously published Finnish registry studies [39], [40] suggesting that the Finnish HLA profile can be estimated with a sample set containing 150 individuals.

One of the limitations of this study was the lack of genome-wide SNP data. Hence, we were not able to use HLA*IMP [58] for allele imputation or analyse the HLA tagging SNPs in the Finnish populations [22]. Because of the differences between populations, population specific validation is highly recommended before using either the HLA*IMP or the HLA tagging SNPs [7], [14], [23].

Taken together, we stress the importance of understanding the population specific MHC haplotypes and the analysis of immunogenetic data. The study of extended *HLA-DRB1* haplotypes indicates the functionality of the implicated genes and provides hypotheses for further assessment of *HLA-DRB1.* The results presented here assist for disease association studies focusing in chronic inflammatory, autoimmune and infectious diseases.

## Supporting Information

Figure S1 **The LD (r^2^) structure of** ***TNF*** **and** ***BTNL2*** **blocks using SNPs.**
(TIF)

Figure S2 **Phylogenetic trees based on the genetic distance of** ***TNF*** **and** ***BTNL2*** **blocks with bootstrap values.**
(TIF)

Figure S3 **A known tag-SNP for** HLA-DRB1*15∶01 **is in strong LD with many other SNPs in the MHC Class III region.** The known proxies are taken from the HapMap [28] and using the software SNAP [38].
(TIF)

Table S1 **The two-locus haplotypes with frequency >1%.**
(DOC)

Table S2 **The observed** ***HLA-DRB1*** **alleles (%) with** ***HLA-A, -B, -DQB1*** **and** ***-DPB1*** **alleles and** ***TNF, C4*** **and** ***BTNL2*** **blocks.**
(DOC)

Table S3 **In the database (HapMap or 1000Genomes) there can be found several proxy SNPs (r^2^>0.9) for the genotyped SNPs.**
(DOC)

Table S4 **A summary of the accepted SNPs (n = 55).** The Finnish allele frequencies were compared with HapMap project (CEU) data [28].
(DOC)

Table S5 **The** ***HLA-DRB1*** **haplotypes with** ***HLA-B*** **alleles, TNF and BTNL2 blocks and C4 allotypes (>1%).**
(DOC)
