# Supplementary figures and images for: Diversity of Extended HLA-DRB1 Haplotypes in the Finnish Population
Source: PLoS One. 2013 Nov 21;8(11):e79690. doi: 10.1371/journal.pone.0079690 (PMC3836878; doi:10.1371/journal.pone.0079690)

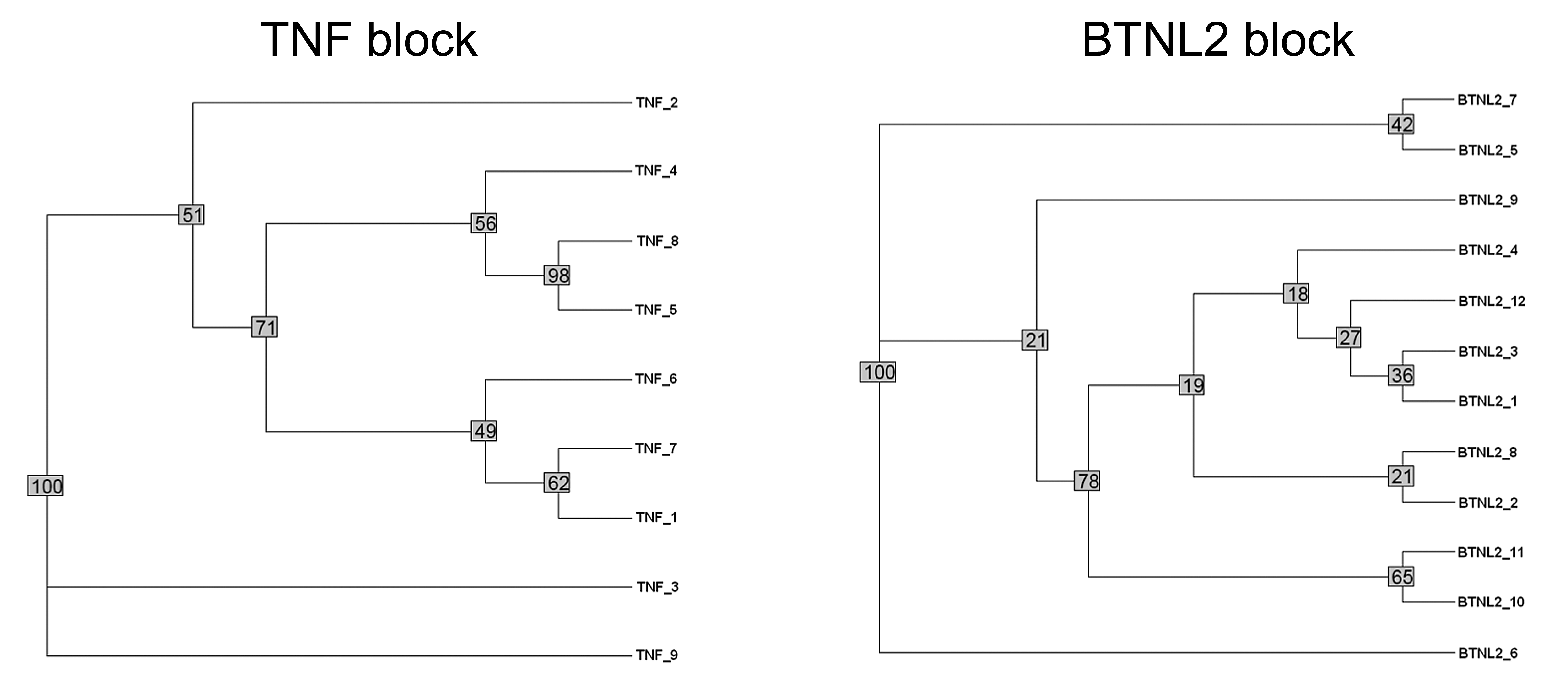

Supplement: Figure S2 — Phylogenetic trees based on the genetic distance of TNF and BTNL2 blocks with bootstrap values. (TIF) [file pone.0079690.s002.tif]

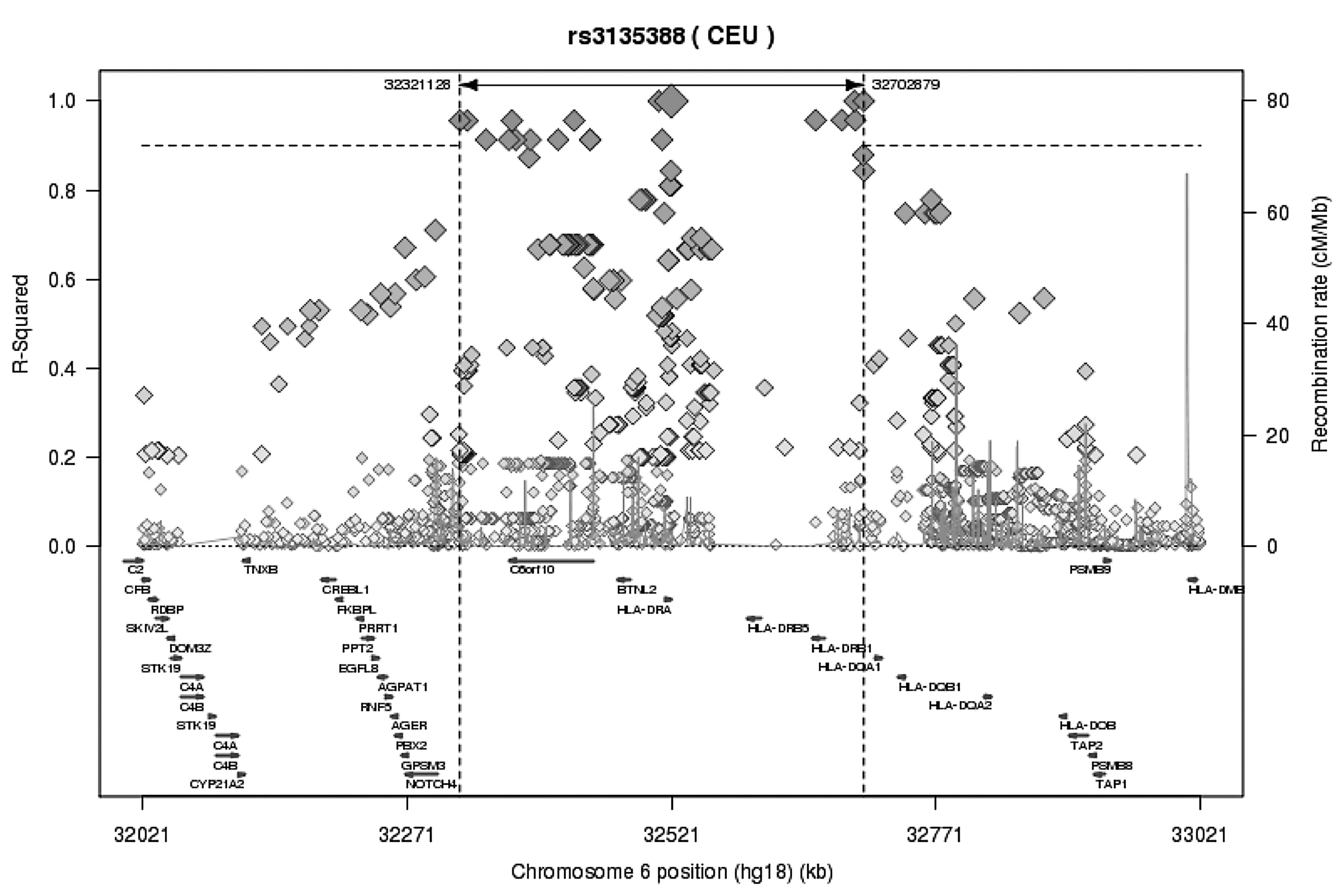

Supplement: Figure S3 — A known tag-SNP for HLA-DRB1*15∶01 is in strong LD with many other SNPs in the MHC Class III region. The known proxies are taken from the HapMap [28] and using the software SNAP [38]. (TIF) [file pone.0079690.s003.tif]
