# Supplementary material for: Diversity of Extended HLA-DRB1 Haplotypes in the Finnish Population
Source: PLoS One. 2013 Nov 21;8(11):e79690. doi: 10.1371/journal.pone.0079690 (PMC3836878; doi:10.1371/journal.pone.0079690)
Supplement: Table S1 — The two-locus haplotypes with frequency >1%. (DOC) [file pone.0079690.s004.doc]

**Table S1**

**The two-locus haplotypes with frequency > 1 %**

| Haplotype DRB1-DPB1 | |  | Haplotype DQB1-DPB1 | |  | Haplotype B-DRB1 | |  |
| --- | --- | --- | --- | --- | --- | --- | --- | --- |
| HLA-DRB1 | HLA-DPB1 | f | HLA-DQB1 | HLA-DPB1 | f | HLA-B | HLA-DRB1 | f |
| *01:01 | *04:02 | 0.058 | *02 | *01:01 | 0.052 | *35 | *01:01 | 0.095 |
| *01:01 | *04:01 | 0.042 | *02 | *04:01 | 0.036 | *07 | *01:01 | 0.019 |
| *01:01 | *02:01 | 0.041 | *02 | *04:02 | 0.019 | *27 | *01:01 | 0.011 |
| *03:01 | *01:01 | 0.047 | *02 | *02:01 | 0.015 | *08 | *03:01 | 0.070 |
| *03:01 | *04:01 | 0.019 | *03:01 | *04:01 | 0.053 | *44 | *03:01 | 0.012 |
| *03:01 | *04:02 | 0.014 | *03:01 | *02:01 | 0.030 | *15 | *04:01 | 0.021 |
| *04:01 | *04:01 | 0.033 | *03:01 | *04:02 | 0.026 | *44 | *04:01 | 0.015 |
| *04:01 | *04:02 | 0.019 | *03:01 | *03:01 | 0.013 | *13 | *07:01 | 0.020 |
| *04:04 | *02:01 | 0.013 | *03:02 | *04:01 | 0.044 | *27 | *08:01 | 0.036 |
| *04:08 | *02:01 | 0.015 | *03:02 | *02:01 | 0.024 | *15 | *08:01 | 0.032 |
| *07:01 | *04:01 | 0.031 | *03:02 | *04:02 | 0.013 | *39 | *08:01 | 0.014 |
| *07:01 | *04:02 | 0.011 | *03:03 | *04:02 | 0.028 | *40 | *08:01 | 0.012 |
| *08:01 | *03:01 | 0.071 | *03:03 | *04:01 | 0.012 | *51 | *09:01 | 0.010 |
| *08:01 | *04:01 | 0.035 | *04 | *03:01 | 0.070 | *44 | *11:01 | 0.012 |
| *08:01 | *04:02 | 0.012 | *04 | *04:01 | 0.036 | *15 | *13:01 | 0.036 |
| *09:01 | *04:02 | 0.021 | *04 | *04:02 | 0.016 | *51 | *13:01 | 0.012 |
| *09:01 | *04:01 | 0.011 | *05:01 | *04:02 | 0.060 | *07 | *13:01 | 0.012 |
| *11:01 | *04:02 | 0.012 | *05:01 | *04:01 | 0.046 | *07 | *15:01 | 0.066 |
| *11:01 | *04:01 | 0.012 | *05:01 | *02:01 | 0.045 | *15 | *15:01 | 0.019 |
| *11:01 | *02:01 | 0.012 | *06:02 | *04:01 | 0.091 | *40 | *13:02 | 0.023 |
| *12:01 | *04:01 | 0.023 | *06:02 | *04:02 | 0.019 | *27 | *04:08 | 0.017 |
| *13:01 | *04:01 | 0.025 | *06:02 | *05:01 | 0.015 |  |  |  |
| *13:01 | *02:01 | 0.024 | *06:02 | *02:01 | 0.011 |  |  |  |
| *13:01 | *05:01 | 0.012 | *06:03 | *04:01 | 0.025 |  |  |  |
| *13:02 | *03:01 | 0.030 | *06:03 | *02:01 | 0.024 |  |  |  |
| *13:02 | *04:02 | 0.013 | *06:03 | *05:01 | 0.012 |  |  |  |
| *15:01 | *04:01 | 0.088 | *06:04 | *03:01 | 0.029 |  |  |  |
| *15:01 | *04:02 | 0.021 | *06:04 | *04:02 | 0.013 |  |  |  |
| *15:01 | *05:01 | 0.014 |  |  |  |  |  |  |

f=frequency
