# Supplementary material for: Diversity of Extended HLA-DRB1 Haplotypes in the Finnish Population
Source: PLoS One. 2013 Nov 21;8(11):e79690. doi: 10.1371/journal.pone.0079690 (PMC3836878; doi:10.1371/journal.pone.0079690)
Supplement: Table S2 — The observed HLA-DRB1 alleles (%) with HLA-A, -B, -DQB1 and -DPB1 alleles and TNF, C4 and BTNL2 blocks. (DOC) [file pone.0079690.s005.doc]

**Table S2**

The observed HLA-DRB1 alleles (%) with HLA-A, -B, -DQB1 and -DPB1 alleles and TNF, C4 and BTNL2 blocks.

| *HLA-A* | *A*01* | *A*02* | *A*03* | *A*11* | *A*24* | *A*26* | *A*29* | *A*31* | *A*32* | *A*68* | Rare  A alleles # | % |
| --- | --- | --- | --- | --- | --- | --- | --- | --- | --- | --- | --- | --- |
| *DRB1*01:01* | 0 | 11 | 68 | 0 | 7 | 0 | 0 | 7 | 7 | 0 | 0 | 100% |
| *DRB1*03:01* | 41 | 7 | 21 | 3 | 3 | 0 | 0 | 0 | 3 | 17 | 3 | 100% |
| *DRB1*04:01* | 5 | 57 | 14 | 0 | 10 | 5 | 0 | 0 | 10 | 0 | 0 | 100% |
| *DRB1*04:03* | 0 | 50 | 0 | 0 | 0 | 0 | 0 | 0 | 50 | 0 | 0 | 100% |
| *DRB1*04:04* | 0 | 50 | 17 | 0 | 0 | 0 | 17 | 0 | 17 | 0 | 0 | 100% |
| *DRB1*04:08* | 0 | 83 | 17 | 0 | 0 | 0 | 0 | 0 | 0 | 0 | 0 | 100% |
| *DRB1*07:01* | 6 | 38 | 25 | 0 | 13 | 6 | 13 | 0 | 0 | 0 | 0 | 100% |
| *DRB1*08:01* | 5 | 57 | 19 | 3 | 8 | 0 | 3 | 0 | 0 | 5 | 0 | 100% |
| *DRB1*09:01* | 0 | 100 | 0 | 0 | 0 | 0 | 0 | 0 | 0 | 0 | 0 | 100% |
| *DRB1*11:01* | 17 | 33 | 8 | 8 | 0 | 0 | 0 | 8 | 8 | 17 | 0 | 100% |
| *DRB1*12:01* | 9 | 73 | 0 | 0 | 0 | 9 | 0 | 0 | 0 | 9 | 0 | 100% |
| *DRB1*13:01* | 4 | 37 | 30 | 4 | 7 | 4 | 0 | 0 | 0 | 11 | 4 | 100% |
| *DRB1*13:02* | 0 | 62 | 8 | 8 | 15 | 0 | 0 | 0 | 0 | 0 | 8 | 100% |
| *DRB1*15:01* | 5 | 45 | 14 | 11 | 9 | 0 | 2 | 7 | 0 | 2 | 5 | 100% |

| *HLA-B* | *B*07* | *B*08* | *B*13* | *B*15* | *B*18* | *B*27* | *B*35* | *B*39* | *B*40* | *B*44* | *B*47* | *B*51* | *B*56* | *B*57* | Rare  B alleles # | % |
| --- | --- | --- | --- | --- | --- | --- | --- | --- | --- | --- | --- | --- | --- | --- | --- | --- |
| *DRB1*01:01* | 14 | 0 | 0 | 2 | 2 | 7 | 64 | 2 | 0 | 2 | 0 | 0 | 2 | 0 | 5 | 100% |
| *DRB1*03:01* | 0 | 79 | 0 | 3 | 0 | 0 | 3 | 0 | 3 | 3 | 3 | 3 | 0 | 0 | 0 | 100% |
| *DRB1*04:01* | 0 | 5 | 5 | 38 | 0 | 0 | 10 | 0 | 10 | 24 | 0 | 0 | 10 | 0 | 0 | 100% |
| *DRB1*04:03* | 0 | 0 | 0 | 0 | 0 | 50 | 0 | 0 | 50 | 0 | 0 | 0 | 0 | 0 | 0 | 100% |
| *DRB1*04:04* | 0 | 0 | 0 | 17 | 0 | 17 | 0 | 0 | 0 | 17 | 0 | 33 | 0 | 0 | 17 | 100% |
| *DRB1*04:08* | 0 | 0 | 0 | 0 | 0 | 83 | 0 | 0 | 0 | 0 | 0 | 0 | 0 | 0 | 17 | 100% |
| *DRB1*07:01* | 6 | 0 | 38 | 13 | 0 | 0 | 6 | 0 | 6 | 13 | 0 | 0 | 0 | 13 | 6 | 100% |
| *DRB1*08:01* | 0 | 5 | 0 | 32 | 0 | 32 | 3 | 8 | 8 | 5 | 0 | 3 | 0 | 0 | 3 | 100% |
| *DRB1*09:01* | 0 | 0 | 0 | 0 | 0 | 10 | 0 | 30 | 20 | 0 | 0 | 30 | 10 | 0 | 0 | 100% |
| *DRB1*11:01* | 8 | 8 | 0 | 17 | 0 | 0 | 0 | 0 | 25 | 25 | 0 | 17 | 0 | 0 | 0 | 100% |
| *DRB1*12:01* | 18 | 0 | 0 | 9 | 0 | 0 | 9 | 9 | 9 | 27 | 0 | 9 | 0 | 0 | 9 | 100% |
| *DRB1*13:01* | 19 | 0 | 0 | 33 | 0 | 7 | 4 | 0 | 0 | 11 | 0 | 15 | 0 | 4 | 7 | 100% |
| *DRB1*13:02* | 8 | 0 | 0 | 0 | 0 | 0 | 0 | 0 | 54 | 15 | 0 | 15 | 8 | 0 | 0 | 100% |
| *DRB1*15:01* | 48 | 7 | 0 | 11 | 7 | 0 | 5 | 2 | 0 | 2 | 7 | 5 | 2 | 2 | 2 | 100% |

| *HLA-DQB1* | *DQB1*02* | *DQB1*03:01* | *DQB1*03:02* | *DQB1*03:03* | *DQB1*04* | *DQB1*05:01* | *DQB1*06:02* | *DQB1*06:03* | *DQB1*06:04* | Rare  DQB1 alleles # | % |
| --- | --- | --- | --- | --- | --- | --- | --- | --- | --- | --- | --- |
| *DRB1*01:01* | 0 | 0 | 0 | 0 | 0 | 100 | 0 | 0 | 0 | 0 | 100% |
| *DRB1*03:01* | 100 | 0 | 0 | 0 | 0 | 0 | 0 | 0 | 0 | 0 | 100% |
| *DRB1*04:01* | 0 | 24 | 76 | 0 | 0 | 0 | 0 | 0 | 0 | 0 | 100% |
| *DRB1*04:03* | 0 | 0 | 100 | 0 | 0 | 0 | 0 | 0 | 0 | 0 | 100% |
| *DRB1*04:04* | 0 | 0 | 100 | 0 | 0 | 0 | 0 | 0 | 0 | 0 | 100% |
| *DRB1*04:08* | 0 | 100 | 0 | 0 | 0 | 0 | 0 | 0 | 0 | 0 | 100% |
| *DRB1*07:01* | 75 | 6 | 0 | 19 | 0 | 0 | 0 | 0 | 0 | 0 | 100% |
| *DRB1*08:01* | 0 | 0 | 0 | 0 | 100 | 0 | 0 | 0 | 0 | 0 | 100% |
| *DRB1*09:01* | 0 | 0 | 0 | 100 | 0 | 0 | 0 | 0 | 0 | 0 | 100% |
| *DRB1*11:01* | 0 | 100 | 0 | 0 | 0 | 0 | 0 | 0 | 0 | 0 | 100% |
| *DRB1*12:01* | 0 | 100 | 0 | 0 | 0 | 0 | 0 | 0 | 0 | 0 | 100% |
| *DRB1*13:01* | 0 | 0 | 0 | 0 | 0 | 0 | 0 | 100 | 0 | 0 | 100% |
| *DRB1*13:02* | 0 | 0 | 0 | 0 | 0 | 0 | 0 | 0 | 100 | 0 | 100% |
| *DRB1*15:01* | 0 | 0 | 0 | 0 | 0 | 0 | 100 | 0 | 0 | 0 | 100% |

| *HLA-DPB1* | *DPB1*01:01* | *DPB1*02:01* | *DPB1*03:01* | *DPB1*04:01* | *DPB1*04:02* | *DPB1*05:01* | Rare DPB1 alleles # | % |
| --- | --- | --- | --- | --- | --- | --- | --- | --- |
| *DRB1*01:01* | 0 | 25 | 2 | 30 | 41 | 2 | 0 | 100% |
| *DRB1*03:01* | 52 | 10 | 7 | 10 | 14 | 0 | 7 | 100% |
| *DRB1*04:01* | 5 | 10 | 10 | 52 | 24 | 0 | 0 | 100% |
| *DRB1*04:03* | 0 | 0 | 25 | 50 | 0 | 25 | 0 | 100% |
| *DRB1*04:04* | 0 | 50 | 0 | 50 | 0 | 0 | 0 | 100% |
| *DRB1*04:08* | 0 | 83 | 0 | 0 | 0 | 0 | 17 | 100% |
| *DRB1*07:01* | 6 | 13 | 0 | 44 | 25 | 0 | 13 | 100% |
| *DRB1*08:01* | 0 | 0 | 57 | 30 | 11 | 0 | 3 | 100% |
| *DRB1*09:01* | 0 | 0 | 0 | 20 | 70 | 0 | 10 | 100% |
| *DRB1*11:01* | 0 | 25 | 8 | 25 | 42 | 0 | 0 | 100% |
| *DRB1*12:01* | 0 | 0 | 18 | 64 | 9 | 9 | 0 | 100% |
| *DRB1*13:01* | 0 | 33 | 4 | 33 | 7 | 7 | 15 | 100% |
| *DRB1*13:02* | 0 | 0 | 69 | 0 | 31 | 0 | 0 | 100% |
| *DRB1*15:01* | 0 | 5 | 5 | 66 | 11 | 11 | 2 | 100% |

| *TNF* block | *TNF_1* | *TNF_2* | *TNF_3* | *TNF_4* | *TNF_5* | *TNF_6* | *TNF_7* | *TNF_8* | *TNF_9* | Rare TNF blocks # | % |
| --- | --- | --- | --- | --- | --- | --- | --- | --- | --- | --- | --- |
| *DRB1*01:01* | 2 | 5 | 2 | 70 | 2 | 2 | 5 | 9 | 0 | 2 | 100% |
| *DRB1*03:01* | 3 | 0 | 83 | 7 | 7 | 0 | 0 | 0 | 0 | 0 | 100% |
| *DRB1*04:01* | 5 | 38 | 10 | 0 | 43 | 0 | 0 | 5 | 0 | 0 | 100% |
| *DRB1*04:03* | 0 | 50 | 0 | 0 | 25 | 0 | 25 | 0 | 0 | 0 | 100% |
| *DRB1*04:04* | 17 | 17 | 0 | 0 | 0 | 0 | 17 | 33 | 0 | 17 | 100% |
| *DRB1*04:08* | 83 | 0 | 0 | 0 | 0 | 17 | 0 | 0 | 0 | 0 | 100% |
| *DRB1*07:01* | 69 | 6 | 6 | 0 | 0 | 0 | 0 | 0 | 0 | 19 | 100% |
| *DRB1*08:01* | 14 | 41 | 5 | 0 | 3 | 24 | 5 | 5 | 3 | 0 | 100% |
| *DRB1*09:01* | 0 | 10 | 0 | 10 | 0 | 80 | 0 | 0 | 0 | 0 | 100% |
| *DRB1*11:01* | 0 | 17 | 33 | 0 | 25 | 8 | 17 | 0 | 0 | 0 | 100% |
| *DRB1*12:01* | 27 | 18 | 0 | 0 | 0 | 18 | 18 | 0 | 0 | 18 | 100% |
| *DRB1*13:01* | 26 | 37 | 15 | 0 | 7 | 0 | 4 | 7 | 0 | 4 | 100% |
| *DRB1*13:02* | 15 | 15 | 0 | 0 | 8 | 0 | 0 | 0 | 62 | 0 | 100% |
| *DRB1*15:01* | 55 | 7 | 7 | 11 | 5 | 2 | 7 | 5 | 0 | 2 | 100% |

| *BTNL2* block | *BTNL2_1* | *BTNL2_2* | *BTNL2_3* | *BTNL2_4* | *BTNL2_5* | *BTNL2_6* | *BTNL2_7* | *BTNL2_8* | *BTNL2_9* | *BTNL2_10* | *BTNL2_11* | *BTNL2_12* | Rare  BTNL2 blocks# | % |
| --- | --- | --- | --- | --- | --- | --- | --- | --- | --- | --- | --- | --- | --- | --- |
| *DRB1*01:01* | 100 | 0 | 0 | 0 | 0 | 0 | 0 | 0 | 0 | 0 | 0 | 0 | 0 | 100% |
| *DRB1*03:01* | 0 | 0 | 0 | 0 | 100 | 0 | 0 | 0 | 0 | 0 | 0 | 0 | 0 | 100% |
| *DRB1*04:01* | 0 | 0 | 95 | 0 | 0 | 0 | 0 | 0 | 0 | 0 | 0 | 0 | 5 | 100% |
| *DRB1*04:03* | 0 | 0 | 100 | 0 | 0 | 0 | 0 | 0 | 0 | 0 | 0 | 0 | 0 | 100% |
| *DRB1*04:04* | 0 | 0 | 50 | 0 | 0 | 0 | 0 | 0 | 0 | 0 | 0 | 0 | 50 | 100% |
| *DRB1*04:08* | 0 | 0 | 100 | 0 | 0 | 0 | 0 | 0 | 0 | 0 | 0 | 0 | 0 | 100% |
| *DRB1*07:01* | 0 | 0 | 44 | 0 | 0 | 0 | 0 | 0 | 0 | 0 | 0 | 25 | 31 | 100% |
| *DRB1*08:01* | 0 | 0 | 0 | 84 | 0 | 0 | 0 | 0 | 0 | 0 | 0 | 0 | 16 | 100% |
| *DRB1*09:01* | 0 | 0 | 0 | 0 | 0 | 0 | 0 | 0 | 0 | 0 | 0 | 0 | 100 | 100% |
| *DRB1*11:01* | 0 | 0 | 0 | 0 | 0 | 0 | 0 | 92 | 0 | 0 | 0 | 0 | 8 | 100% |
| *DRB1*12:01* | 0 | 0 | 0 | 0 | 0 | 0 | 0 | 0 | 0 | 55 | 45 | 0 | 0 | 100% |
| *DRB1*13:01* | 0 | 0 | 0 | 0 | 0 | 59 | 0 | 0 | 11 | 0 | 0 | 0 | 30 | 100% |
| *DRB1*13:02* | 0 | 0 | 0 | 0 | 0 | 0 | 92 | 0 | 0 | 0 | 0 | 0 | 8 | 100% |
| *DRB1*15:01* | 0 | 98 | 0 | 0 | 0 | 0 | 0 | 0 | 0 | 0 | 0 | 0 | 2 | 100% |

| *C4* block | *C4_1* | *C4_2* | *C4_3* | *C4_4* | *C4_5* | *C4_6* | Rare  C4 blocks# | % |
| --- | --- | --- | --- | --- | --- | --- | --- | --- |
| *DRB1*01:01* | 20 | 64 | 16 | 0 | 0 | 0 | 0 | 100% |
| *DRB1*03:01* | 24 | 3 | 0 | 72 | 0 | 0 | 0 | 100% |
| *DRB1*04:01* | 52 | 38 | 10 | 0 | 0 | 0 | 0 | 100% |
| *DRB1*04:03* | 75 | 0 | 25 | 0 | 0 | 0 | 0 | 100% |
| *DRB1*04:04* | 100 | 0 | 0 | 0 | 0 | 0 | 0 | 100% |
| *DRB1*04:08* | 100 | 0 | 0 | 0 | 0 | 0 | 0 | 100% |
| *DRB1*07:01* | 88 | 0 | 0 | 6 | 0 | 0 | 6 | 100% |
| *DRB1*08:01* | 68 | 0 | 32 | 0 | 0 | 0 | 0 | 100% |
| *DRB1*09:01* | 90 | 0 | 10 | 0 | 0 | 0 | 0 | 100% |
| *DRB1*11:01* | 92 | 0 | 0 | 8 | 0 | 0 | 0 | 100% |
| *DRB1*12:01* | 73 | 18 | 0 | 0 | 0 | 9 | 0 | 100% |
| *DRB1*13:01* | 48 | 33 | 4 | 4 | 0 | 11 | 0 | 100% |
| *DRB1*13:02* | 31 | 0 | 0 | 0 | 69 | 0 | 0 | 100% |
| *DRB1*15:01* | 80 | 0 | 5 | 2 | 2 | 11 | 0 | 100% |

# All the rare combinations between HLA-DRB1 and other alleles or blocks grouped

Only HLA-DRB1 alleles n > 3 presented here. The HLA-DRB1 allele counts (n) are shown in Table 1
