# Supplementary material for: Diversity of Extended HLA-DRB1 Haplotypes in the Finnish Population
Source: PLoS One. 2013 Nov 21;8(11):e79690. doi: 10.1371/journal.pone.0079690 (PMC3836878; doi:10.1371/journal.pone.0079690)
Supplement: Table S3 — In the database (HapMap or 1000Genomes) there can be found several proxy SNPs (r2>0.9) for the genotyped SNPs. (DOC) [file pone.0079690.s006.doc]

**Table S3**

**In the database (HapMap or 1000Genomes) there can be found several proxy SNPs (r^2^>0.9) for the genotyped SNPs**.

|  | **HapMap Release 22** | | **HapMap Release 3** | | **1000Genomes Pilot** | |
| --- | --- | --- | --- | --- | --- | --- |
| Genotyped SNP | Proxy | GeneVariant | Proxy | GeneVariant | Proxy |  |
| **rs2239704** |  | | | | | |
| **5'** | rs2844484 | upstream | rs2857602 | intergenic | rs116137681 |  |
|  | rs2857602 | intergenic | rs928815 | downstream | rs116061304 |  |
|  | rs928815 | downstream | rs2516390 | downstream | rs114263655 |  |
|  | rs2516390 | downstream |  |  | rs116608786 |  |
|  | rs2516479 | downstream |  |  | rs116060750 |  |
|  |  |  |  |  | rs114465669 |  |
|  |  |  |  |  | rs116665275 |  |
|  |  |  |  |  | rs116592789 |  |
|  |  |  |  |  | rs115488032 |  |
|  |  |  |  |  |  |  |
| **rs2076530** |  | | | | | |
| **Non-Synonymous** | rs2076529 | synonymous, coding, intergenic | rs2076529 | synonymous, coding, intergenic |  |  |
|  | rs2076533 | intronic, intergenic | rs9268472 | upstream, intergenic |  |  |
|  | rs3817973 | downstream, intergenic | rs3817973 | downstream, intergenic |  |  |
|  | rs9268473 | upstream, intergenic | rs4424066 | upstream, intergenic |  |  |
|  | rs4424066 | upstream, intergenic | rs9268460 | intronic, intergenic |  |  |
|  | rs2395153 | intronic, intergenic | rs2076533 | intronic, intergenic |  |  |
| **rs3135388** |  | | | | | |
| **downstream** | rs3129889 | downstream | rs3129889 | downstream |  |  |
|  | rs3135391 | synonymous, coding | rs3135391 | synonymous, coding |  |  |
|  | rs3129860 | intergenic | rs3135350 | intergenic |  |  |
|  | rs3135005 | intergenic | rs3135352 | intergenic |  |  |
|  | rs3104391 | intergenic | rs9271366 | intergenic |  |  |
|  | rs701831 | N/A | rs3129868 | upstream |  |  |
|  | rs9270986 | intergenic | rs9270984 | intergenic |  |  |
|  | rs9271366 | intergenic | rs9270986 | intergenic |  |  |
|  | rs3117117 | intronic | rs9271055 | intergenic |  |  |
|  | rs6457535 | intronic | rs3117116 | intronic, intergenic |  |  |
|  | rs9267992 | upstream, intergenic |  |  |  |  |
|  | rs9267955 | intergenic |  |  |  |  |
|  | rs3129868 | upstream |  |  |  |  |
|  | rs3129938 | intronic |  |  |  |  |
|  | rs3129934 | intronic |  |  |  |  |
|  | rs3129900 | intronic |  |  |  |  |
|  | rs9268205 | intronic |  |  |  |  |
|  | rs9268154 | intronic |  |  |  |  |
|  | rs9268148 | downstream |  |  |  |  |
|  | rs6913182 | intergenic |  |  |  |  |
| **rs2213585** |  | | | | | |
| **downstream** | rs2213586 | downstream | rs2213586 | downstream |  |  |
|  | rs7195 | 3' | rs2227139 | downstream |  |  |
|  | rs7194 | 3' | rs7195 | 3' |  |  |
|  | rs7192 | Non-Synonymous, coding | rs7194 | 3' |  |  |
|  | rs2227139 | downstream | rs7192 | Non-Synonymous, coding |  |  |
|  | rs3763327 | downstream | rs7754768 | intergenic |  |  |
|  | rs9268832 | Non-Synonymous, coding, intergenic | rs9268832 | Non-Synonymous, coding, intergenic |  |  |
