# Supplementary material for: Diversity of Extended HLA-DRB1 Haplotypes in the Finnish Population
Source: PLoS One. 2013 Nov 21;8(11):e79690. doi: 10.1371/journal.pone.0079690 (PMC3836878; doi:10.1371/journal.pone.0079690)
Supplement: Table S4 — A summary of the accepted SNPs (n = 55). The Finnish allele frequencies were compared with HapMap project (CEU) data [28]. (DOC) [file pone.0079690.s007.doc]

**Table S4**

A summary of the accepted SNPs (n=55). The Finnish allele frequencies were compared with HapMap project (CEU) data[28].

|  | | | | Finnish healthy subjects (n=150) | | | | | | | HapMap CEU | | | |  |
| --- | --- | --- | --- | --- | --- | --- | --- | --- | --- | --- | --- | --- | --- | --- | --- |
| SNPs in TNF Block | Position (hg18) | Gene (s) | Predicted function | ObsHET | PredHET | HWE | Allele 1 | f | Allele 2 | f | Allele 1 | f | Allele 2 | f | P value |
| rs2009658 | 31538244 | LTA | 5upstream | 0.22 | 0.23 | 0.96 | C | 0.87 | G | 0.13 | C | 0.84 | G | 0.16 |  |
| rs2239704 | 31540141 | LTA | 5utr | 0.40 | 0.46 | 0.21 | C | 0.65 | A | 0.35 | C | 0.58 | A | 0.42 |  |
| rs2229094 | 31540556 | LTA | coding | 0.35 | 0.41 | 0.15 | T | 0.72 | C | 0.28 | T | 0.75 | C | 0.25 |  |
| rs2229092 | 31540757 | LTA | coding | 0.08 | 0.09 | 0.54 | A | 0.95 | C | 0.05 | A | 0.94 | C | 0.06 |  |
| rs1041981 | 31540784 | LTA | coding | 0.46 | 0.47 | 0.92 | C | 0.63 | A | 0.37 | C | 0.66 | A | 0.34 |  |
| rs1799964 | 31542308 | LTA/TNF | 3downstream, 5upstream | 0.25 | 0.25 | 1.00 | T | 0.86 | C | 0.14 | T | 0.80 | C | 0.20 |  |
| rs1799724 | 31542482 | LTA/TNF | 3downstream, 5upstream | 0.11 | 0.11 | 0.84 | C | 0.94 | T | 0.06 |  |  |  |  | # |
| rs1800629 | 31543031 | LTA/TNF | 3downstream, 5upstream | 0.25 | 0.26 | 0.94 | G | 0.85 | A | 0.15 | G | 0.82 | A | 0.18 |  |
| rs361525 | 31543101 | LTA/TNF | 3downstream, 5upstream | 0.03 | 0.03 | 1.00 | G | 0.99 | A | 0.01 |  |  |  |  | # |
| rs3093664 | 31544642 | TNF | intronic | 0.09 | 0.08 | 1.00 | A | 0.96 | G | 0.04 |  |  |  |  | # |
| rs769178 | 31547514 | TNF/LTB | 3downstream | 0.10 | 0.11 | 0.78 | G | 0.94 | T | 0.06 | G | 0.94 | T | 0.06 |  |
| rs3093553 | 31549556 | LTB | intronic | 0.05 | 0.05 | 1.00 | T | 0.98 | G | 0.02 | T | 0.93 | G | 0.07 |  |
| rs2256965 | 31555130 | LST1/NCR3 | intronic | 0.47 | 0.49 | 0.70 | G | 0.58 | A | 0.42 | G | 0.56 | A | 0.44 |  |

|  | | | | Finnish healthy subjects (n=150) | | | | | | | HapMap CEU | | | |  |
| --- | --- | --- | --- | --- | --- | --- | --- | --- | --- | --- | --- | --- | --- | --- | --- |
| SNPs in BTNL2 block | Position (hg18) | Gene (s) | Predicted function | ObsHET | PredHET | HWE | Allele 1 | f | Allele 2 | f | Allele 1 | f | Allele 2 | f | P value |
| rs28362678 | 32362745 | BTNL2 | coding | 0.29 | 0.34 | 0.12 | C | 0.78 | T | 0.22 |  |  |  |  | # |
| rs2076530 | 32363816 | BTNL2 | coding | 0.47 | 0.48 | 1.00 | T | 0.61 | C | 0.39 | T | 0.53 | C | 0.47 |  |
| rs9268480 | 32363844 | BTNL2 | coding | 0.29 | 0.28 | 1.00 | C | 0.83 | T | 0.17 | C | 0.65 | T | 0.35 | 0.006 |
| rs2076529 | 32363955 | BTNL2 | coding | 0.46 | 0.48 | 0.78 | T | 0.61 | C | 0.39 | T | 0.53 | C | 0.47 |  |
| rs3793127 | 32371915 | BTNL2 | intronic | 0.23 | 0.30 | 0.01 | C | 0.82 | T | 0.18 | C | 0.74 | T | 0.26 |  |
| rs28362683 | 32372963 | BTNL2 | coding | 0.28 | 0.31 | 0.44 | G | 0.81 | A | 0.19 | G | 0.95 | A | 0.05 | 0.004 |
| rs3763311 | 32376176 | BTNL2 | 5upstream | 0.30 | 0.31 | 0.90 | C | 0.81 | T | 0.19 | C | 0.65 | T | 0.35 | 0.016 |
| rs3763312 | 32376348 | BTNL2 | 5upstream | 0.27 | 0.26 | 0.98 | G | 0.84 | A | 0.16 | G | 0.73 | A | 0.27 |  |
| rs3763313 | 32376471 | BTNL2 | 5upstream | 0.41 | 0.42 | 0.87 | A | 0.70 | C | 0.30 | A | 0.81 | C | 0.19 |  |
| rs3763317 | 32376788 | BTNL2 | 5upstream | 0.51 | 0.50 | 1.00 | C | 0.51 | T | 0.49 | C | 0.52 | T | 0.48 |  |
| rs5007259 | 32379101 | BTNL2 | 5upstream | 0.50 | 0.50 | 1.00 | T | 0.50 | C | 0.50 | T | 0.43 | C | 0.57 |  |
| rs17208888 | 32379506 | BTNL2 | 5upstream | 0.11 | 0.12 | 0.91 | G | 0.94 | A | 0.06 | G | 0.93 | A | 0.07 |  |
| rs9405098 | 32379736 | BTNL2 | 5upstream | 0.05 | 0.06 | 0.20 | G | 0.97 | A | 0.03 | G | 0.98 | A | 0.02 |  |
| rs9268528 | 32383108 | BTNL2 |  | 0.37 | 0.41 | 0.24 | A | 0.71 | G | 0.29 | A | 0.60 | G | 0.40 |  |
| rs9268541 | 32384527 | BTNL2 |  | 0.08 | 0.09 | 0.54 | T | 0.95 | C | 0.05 | T | 0.95 | C | 0.05 |  |
| rs2395166 | 32388275 | BTNL2 |  | 0.46 | 0.45 | 0.89 | T | 0.66 | C | 0.34 | T | 0.55 | C | 0.45 |  |
| rs3135365 | 32389255 | BTNL2 |  | 0.30 | 0.27 | 0.40 | T | 0.84 | G | 0.16 | **T** | **0.72** | **G** | **0.28** |  |
| rs3135363 | 32389648 | BTNL2 |  | 0.41 | 0.41 | 1.00 | T | 0.71 | C | 0.29 | **T** | **0.81** | **C** | **0.19** |  |
| rs3135351 | 32392945 | BTNL2 |  | 0.32 | 0.34 | 0.52 | G | 0.78 | T | 0.22 | **G** | **0.90** | **T** | **0.10** | 0.033 |
| rs3135344 | 32395036 | BTNL2/ DRA |  | 0.42 | 0.44 | 0.63 | A | 0.67 | G | 0.33 | **A** | **0.64** | **G** | **0.36** |  |
| rs3129843 | 32395726 | BTNL2/ DRA |  | 0.18 | 0.18 | 1.00 | A | 0.90 | G | 0.10 | A | 0.92 | G | 0.08 |  |
| rs3135341 | 32398748 | BTNL2/ DRA |  | 0.30 | 0.28 | 0.40 | T | 0.83 | G | 0.17 | **T** | **0.75** | **G** | **0.25** |  |
| rs2027856 | 32402705 | BTNL2/ DRA |  | 0.20 | 0.23 | 0.19 | C | 0.87 | T | 0.13 | **C** | **0.90** | **T** | **0.10** |  |
| rs3129871 | 32406342 | HLA-DRA | 5upstream | 0.47 | 0.48 | 0.78 | C | 0.59 | A | 0.41 | C | 0.63 | A | 0.38 |  |
| rs9405035 | 32407068 | HLA-DRA | 5upstream | 0.06 | 0.07 | 0.35 | G | 0.96 | A | 0.04 | G | 0.98 | A | 0.02 |  |
| rs9268644 | 32408044 | HLA-DRA | intronic | 0.38 | 0.49 | 0.01 | A | 0.56 | C | 0.44 | A | 0.42 | C | 0.58 |  |
| rs3129877 | 32408597 | HLA-DRA | intronic | 0.36 | 0.47 | 0.01 | G | 0.62 | A | 0.38 | G | 0.77 | A | 0.23 | 0.031 |
| rs3135392 | 32409242 | HLA-DRA | intronic | 0.45 | 0.49 | 0.32 | T | 0.56 | G | 0.44 | **T** | **0.32** | **G** | **0.68** | 0.001 |
| rs3129882 | 32409530 | HLA-DRA | intronic | 0.47 | 0.49 | 0.60 | A | 0.56 | G | 0.44 | A | 0.58 | G | 0.42 |  |
| rs8084 | 32411035 | HLA-DRA | coding | 0.44 | 0.49 | 0.22 | A | 0.56 | C | 0.44 | A | 0.40 | C | 0.60 | 0.033 |
| rs2239804 | 32411523 | HLA-DRA | intronic | 0.35 | 0.41 | 0.15 | A | 0.72 | G | 0.28 | **A** | **0.51** | **G** | **0.49** | 0.004 |
| rs11544315 | 32411573 | HLA-DRA | coding | 0.03 | 0.03 | 1.00 | G | 0.99 | A | 0.01 | **G** | **0.95** | **A** | **0.05** |  |
| rs3177928 | 32412435 | HLA-DRA | 3downstream | 0.23 | 0.28 | 0.07 | G | 0.83 | A | 0.17 | G | 0.83 | A | 0.17 |  |
| rs3135388 | 32413051 | HLA-DRA | 3downstream | 0.27 | 0.26 | 0.63 | C | 0.85 | T | 0.15 | **C** | **0.82** | **T** | **0.18** |  |
| rs2213585 | 32413150 | HLA-DRA | 3downstream | 0.43 | 0.50 | 0.11 | C | 0.55 | T | 0.45 | **C** | **0.34** | **T** | **0.66** | 0.004 |
| rs6937545 | 32418031 | HLA-DRA |  | 0.44 | 0.47 | 0.45 | C | 0.61 | A | 0.39 | C | 0.69 | A | 0.31 |  |
| rs9268833 | 32428062 | HLA-DRB9 | non-coding intronic | 0.23 | 0.30 | 0.02 | C | 0.82 | T | 0.18 | C | 0.63 | T | 0.37 | 0.004 |
| rs6919855 | 32429011 | HLA-DRB9 | non-coding intronic | 0.37 | 0.36 | 0.81 | T | 0.77 | C | 0.23 | T | 0.69 | C | 0.31 |  |
| rs7766843 | 32430729 | HLA-DRB9 | non-coding intronic | 0.44 | 0.48 | 0.41 | C | 0.61 | T | 0.39 | C | 0.84 | T | 0.16 | 0.000 |
| rs2395185 | 32433167 | HLA-DRB9 | non-coding intronic | 0.29 | 0.34 | 0.09 | G | 0.78 | T | 0.22 | G | 0.58 | T | 0.42 | 0.004 |
| rs9268979 | 32435044 | HLA-DRB9 | non-coding intronic | 0.49 | 0.48 | 0.95 | C | 0.61 | T | 0.39 | C | 0.57 | T | 0.43 |  |
| rs7748472 | 32448763 | HLA-DRB9/ DRB5 | | 0.07 | 0.08 | 0.48 | A | 0.96 | G | 0.04 | A | 0.98 | G | 0.02 |  |

# not genotyped in the HapMap project (combined Phase II and III data, release 27)

The SNPs that are bolded were originally genotyped in other strand in the HapMap project

ObsHET = observed heterozygosity

PredHET=predicted heterozygosity

HWE = Hardy -Weinberg Equilibrium
