# Supplementary material for: Diversity of Extended HLA-DRB1 Haplotypes in the Finnish Population
Source: PLoS One. 2013 Nov 21;8(11):e79690. doi: 10.1371/journal.pone.0079690 (PMC3836878; doi:10.1371/journal.pone.0079690)
Supplement: Table S5 — The HLA-DRB1 haplotypes with HLA-B alleles, TNF and BTNL2 blocks and C4 allotypes (>1%). (DOC) [file pone.0079690.s008.doc]

**Table S5**

The HLA-DRB1 haplotypes with HLA-B alleles, TNF and BTNL2 blocks and C4 allotypes (>1 %)

|  | | | C4 allotypes | |  | |
| --- | --- | --- | --- | --- | --- | --- |
| DRB1 | HLA-B | TNF | C4A | C4B | BTNL2 | f |
| **01:01* | **35* | *TNF_4* | 3 | 0, conv A2 | *BTNL2_1* | 0.050 |
| **01:01* | **35* | *TNF_4* | 2 | Q0 | *BTNL2_1* | 0.016 |
| **03:01* | **08* | *TNF_3* | Q0 | 1 | *BTNL2_5* | 0.059 |
| **03:01* | **08* | *TNF_3* | 3 | 1 | *BTNL2_5* | 0.011 |
| **04:01* | **15* | *TNF_5* | 3 | 2 | *BTNL2_3* | 0.013 |
| **07:01* | **13* | *TNF_6* | 3 | 1 | *BTNL2_3* | 0.020 |
| **08:01* | **15* | *TNF_6* | 3 | 1 | *BTNL2_4* | 0.020 |
| **08:01* | **27* | *TNF_2* | 3 | Q0 | *BTNL2_4* | 0.019 |
| **08:01* | **15* | *TNF_2* | 3 | 1 | *BTNL2_4* | 0.013 |
| **13:02* | **40* | *TNF_9* | InsCT | 2 | *BTNL2_7* | 0.023 |
| **15:01* | **07* | *TNF_1* | 3 | 1 | *BTNL2_2* | 0.045 |

f=haplotype frequency

conv A2= C4B gene converted into C4A gene (C4 2 allotype)

Q0 = C4 deficiency

InsCT = insertion in C4A gene
